# A high density linkage map of the bovine genome

**DOI:** 10.1186/1471-2156-10-18

**Published:** 2009-04-24

**Authors:** Juan A Arias, Mike Keehan, Paul Fisher, Wouter Coppieters, Richard Spelman

**Affiliations:** 1 Livestock Improvement Corporation, Private Bag 3016, Hamilton 3240, New Zealand; 2 Animal Genomics, Université de Liège, Liège, Belgium

## Abstract

**Background:**

Recent technological advances have made it possible to efficiently genotype large numbers of single nucleotide polymorphisms (SNPs) in livestock species, allowing the production of high-density linkage maps. Such maps can be used for quality control of other SNPs and for fine mapping of quantitative trait loci (QTL) via linkage disequilibrium (LD).

**Results:**

A high-density bovine linkage map was constructed using three types of markers. The genotypic information was obtained from 294 microsatellites, three milk protein haplotypes and 6769 SNPs. The map was constructed by combining genetic (linkage) and physical information in an iterative mapping process. Markers were mapped to 3,155 unique positions; the 6,924 autosomal markers were mapped to 3,078 unique positions and the 123 non-pseudoautosomal and 19 pseudoautosomal sex chromosome markers were mapped to 62 and 15 unique positions, respectively. The linkage map had a total length of 3,249 cM. For the autosomes the average genetic distance between adjacent markers was 0.449 cM, the genetic distance between unique map positions was 1.01 cM and the average genetic distance (cM) per Mb was 1.25.

**Conclusion:**

There is a high concordance between the order of the SNPs in our linkage map and their physical positions on the most recent bovine genome sequence assembly (Btau 4.0). The linkage maps provide support for fine mapping projects and LD studies in bovine populations. Additionally, the linkage map may help to resolve positions of unassigned portions of the bovine genome.

## Background

Advances in technology have dramatically increased the ability to cost-effectively genotype a large number of SNPs in humans and farm animals [1,2]. The majority of the SNPs have been placed in physical, but not linkage maps. Increasing the resolution of bovine linkage maps will improve estimates of linkage disequilibrium (LD) [3,4] and increase the success rate of fine mapping quantitative trait loci (QTL) in cattle. The possibility that any particular SNP does not have a functional role is outweighed by its indirect use as a genetic marker associated to a causal variant [5]. In addition, mapped SNPs provide information about LD patterns over the genome and allow the identification of haplotype blocks [4,6,7].

Historically a diverse variety of methodologies and procedures have been used to order bovine chromosomal segments [8-16]. A physical map [17] and several linkage maps have been reported for the bovine genome [16,18-22]. To date, the linkage map of Snelling et al. [16] has the highest number of genetic markers positioned. Their linkage map is comprised of 4,585 markers (including 913 SNPs), in 2,475 unique positions covering 3,058 centimorgans (cM) in total. Since Kappes et al. [23] reported advances in the sequencing of the bovine genome, a 7.1 fold coverage of the genome has been attained and this has generated over 2 million bovine SNPs that are currently in NCBI dbSNP Build 129 [24]. Affymetrix produced a commercial genotyping panel of approximately 10,000 bovine SNPs [25], 92% of which were derived from this sequencing resource [26]; the remaining eight percent were derived from Australia's Commonwealth Scientific and Industrial Research Organisation (CSIRO) [27].

The objective of this work is to present a high-density bovine linkage map (HDBLM) that combines a low-density microsatellite based linkage map (LDM) with SNPs from the Affymetrix GeneChip™ Bovine Mapping 10K SNP kit (hereafter called 10K SNP panel) [25]. Results from the HDBLM could enhance the understanding of the alignment and orientation of contigs and scaffolds in the bovine genome assembly, thus allowing the examination of relationships between physical distances, linkage disequilibrium (LD) and genetic map distance. This would provide a framework to identify causal relationships between genomic variation and animal performance traits.

## Results

### Genotype quality

Genotypes were received from Affymetrix (Santa Clara CA, USA) for 9,713 SNPs with an average call rate of 99.25% for the 10K SNP panel. A total of 1,891 SNPs were removed for the following reasons: departure from Hardy-Weinberg Equilibrium (HWE) (120), more than 50 inheritance inconsistencies (260), having an allele with frequency lower than 5% (1,494), and less than 10 informative meioses (17) (Additional file 1). Genotypes from six animals were used as blind duplicates with an average concordance between of samples of 99.93%. A total of 1,189 SNPs (hereafter called orphan SNPs) were not initially assigned to any one chromosome; 1,053 of these SNPs were subsequently assigned to a single chromosome. There were 955 SNPs from the 10K SNP panel initially incorrectly assigned to a chromosome (hereafter called displaced SNPs), 779 of which we were able to re-assign to a different chromosome. The stringent threshold criteria utilized for the assignment of these SNPs prevented the allocation of some of the 136 orphan SNPs and some of the 176 displaced SNPs. The inability to place to a chromosome some of these orphan and displaced SNPs could have been reduced by lowering the stringency of the threshold criteria used during the assignment. The final marker data set consisted of 7,510 SNPs from the 10K SNP panel in addition to 294 microsatellites, three milk protein haplotypes and two gene-based SNPs.

### Genetic maps

Table 1 shows the mean number of informative meioses for all of the autosomal markers. The method of Breen et al. [28] was used to calculate the resolution for an autosomal marker map. Using the average of 366.9 informative meioses, the 95% confidence level for a distance was calculated to be 0.80 cM.

**Table 1:** Informative meioses for autosomal chromosomes

**Marker Type**	**N^a^**	**Mean^b^**	**Standard Deviation**	**Minimum**	**Maximum**
SNP^c^	6634	349.3	158.4	10	800
Microsatellite^d^	285	778.5	219.3	112	1237
Haplotype^e^	3	278.7	148.6	133	430
Other^f^	2	146.5	177.5	21	272
					
All	6924	366.9	182.5	10	1237

a Number of markers.

b Mean of informative meioses.

c From the 10K SNP panel.

d Description in Additional file 2.

e Milk protein haplotypes (1) Alpha s1 casein (CSN1S1): A_CAS_41_26, AS_CAS_192; 2) Kappa casein (CSN3): K_CAS_148, and 3) Beta casein (CSN2): B_CAS_37, B_CAS_67, B_CAS_106 and B_CAS_122

f Gene-based SNPs (1) DGAT1: K232A and 2) GHR: F279Y.

**Figure 1:**
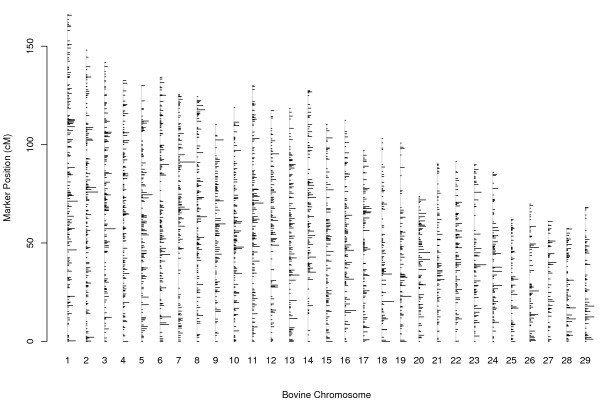
**Marker locations on bovine genome sequence autosomes**. Linkage maps for bovine autosomal chromosomes are presented here. Vertical lines symbolize bovine chromosomes. Horizontal lines on vertical lines represent locations of markers in chromosomes. Length of horizontal lines is proportional to number of markers at same location.

A total of 7,066 markers were mapped (294 microsatellites, three haplotypes and 6,769 SNPs) (Table 2). The autosomal markers were distributed across 3,078 unique positions (Figure 1). The linkage map for the 29 bovine autosomal chromosomes was 3,097.4 cM with an average Kosambi distance [29] of 0.449 cM. The smallest genetic distance present in each chromosome was 0 cM and the largest genetic distance was 8.7 cM, on chromosome 14. The 123 markers mapped to the non-pseudoautosomal region covered 105.8 cM and the 19 markers mapped to the pseudoautosomal region covered 45.3 cM. These regions were mapped separately. The maximum genetic distance was 9.3 cM for the non-pseudoautosomal region and 9.7 cM for the pseudoautosomal chromosome region. The smallest and largest average genetic distance over an individual chromosome was 0.38 cM and 0.59 cM for chromosomes 28 and 21, respectively. Chromosome 25 had the lowest coefficient of variation (CV) for genetic distance (1.51) and chromosome 14 the highest CV for genetic distance (2.13) (Table 2).

**Table 2:** Description of linkage maps

Chr^a^	N^b^	N SNPs^c^	Unique positions	Length^d ^(cM)	Mean rec. dist.^e ^(cM)	S. dev. rec. dist.^f^	Minimum rec. dist.^g ^(cM)	Maximum rec. dist.^h ^(cM)	C. var. rec. dist.^i^
1	412	395	184	166.0	0.40	0.77	0	5.70	1.91
2	325	316	163	148.0	0.46	0.78	0	4.86	1.71
3	312	304	152	141.8	0.46	0.80	0	5.16	1.76
4	303	286	150	132.5	0.44	0.74	0	3.92	1.68
5	315	305	125	130.0	0.41	0.83	0	7.80	2.01
6	318	305	140	134.2	0.42	0.80	0	5.20	1.89
7	282	271	138	125.5	0.45	0.84	0	7.49	1.87
8	284	275	128	124.4	0.44	0.76	0	5.70	1.72
9	236	227	99	110.3	0.47	0.87	0	5.50	1.85
10	287	270	127	118.9	0.41	0.78	0	7.50	1.89
11	311	304	143	129.9	0.42	0.73	0	6.30	1.74
12	239	232	97	117.3	0.49	0.94	0	5.50	1.91
13	277	271	117	118.3	0.43	0.78	0	4.70	1.81
14	271	248	125	127.4	0.47	1.01	0	8.66	2.13
15	231	223	98	110.3	0.48	0.83	0	4.90	1.73
16	257	238	112	112.4	0.44	0.82	0	5.90	1.86
17	230	215	100	97.0	0.42	0.76	0	4.30	1.81
18	190	184	82	103.2	0.55	1.04	0	7.70	1.91
19	176	168	76	100.8	0.58	1.18	0	7.40	2.04
20	222	216	79	73.7	0.33	0.62	0	3.30	1.86
21	154	146	82	90.2	0.59	0.90	0	5.40	1.53
22	203	196	87	91.4	0.45	0.87	0	5.10	1.94
23	181	164	71	90.0	0.50	1.00	0	7.60	1.99
24	202	196	88	85.8	0.43	0.79	0	3.90	1.84
25	122	117	64	62.0	0.51	0.77	0	3.70	1.51
26	161	156	67	69.8	0.44	0.83	0	6.00	1.89
27	125	120	52	60.9	0.49	0.89	0	3.80	1.82
28	154	149	68	57.3	0.38	0.67	0	3.70	1.77
29	144	137	64	68.0	0.48	0.95	0	6.30	2.00
X^j^	123	116	62	105.8	2.51	2.77	0	9.35	1.10
X(Y)^k^	19	17	15	45.3	0.87	1.37	0	9.70	1.57
									
ALL^l^	6924	6634	3078	3097.4	0.46	0.84	0	8.66	1.84

a Chromosome number.

b Number of markers.

c Number of SNPs.

d Chromosome linkage map length.

e Mean Kosambi distance (Kosambi, 1944).

f Standard deviation for Kosambi distance.

g Minimum recombination distance.

h Maximum recombination distance.

i Coefficient of variation recombination distances.

j Non-pseudoautosomal region.

k Pseudoautosomal region.

l Autosomal

On average the genetic distance per unit of physical distance (cM/Mb) was 1.25 (Table 3). Chromosome 20 had the lowest cM per Mb ratio. Chromosome size accounted for 42% of the variation in inter-chromosomal genetic distances per Mb (P-value 6.5 × 10^-5^); the correlation of chromosome size to recombination distance was -0.66.

**Table 3:** Average recombination distance per Mb

Chromosome	Linkage map (cM)	Physical map (Mb)^a^	cM/Mb^b^	Pearson correlation^c^
1	166.0	160.8	1.03	0.996
2	148.0	139.0	1.07	0.988
3	141.8	126.1	1.12	0.961
4	132.5	123.7	1.07	0.997
5	130.0	124.8	1.04	0.993
6	134.2	122.5	1.10	0.994
7	125.5	111.7	1.12	0.986
8	124.4	116.7	1.07	0.996
9	110.3	107.1	1.03	0.989
10	118.9	103.9	1.14	0.993
11	129.9	109.6	1.19	0.993
12	117.3	84.8	1.38	0.981
13	118.3	83.7	1.41	0.984
14	127.4	81.1	1.57	0.988
15	110.3	81.0	1.36	0.990
16	112.4	77.8	1.44	0.987
17	97.0	75.0	1.29	0.997
18	103.2	65.8	1.57	0.994
19	100.8	63.8	1.58	0.985
20	73.7	73.8	1.00	0.996
21	90.2	66.8	1.35	0.982
22	91.4	59.9	1.53	0.991
23	90.0	53.1	1.69	0.937
24	85.8	64.9	1.32	0.993
25	62.0	42.9	1.45	0.999
26	69.8	51.7	1.35	0.987
27	60.9	48.7	1.25	0.993
28	57.3	45.1	1.27	0.996
29	68.0	51.5	1.32	0.995
X^d^	105.8	82.7	1.28	0.92
X(Y)^e^	45.3	54.7	0.82	0.67
				
Total	3248.5	2605.7	1.25	0.985

a Bovine genome sequence assembly (Btau 4.0).

b Recombination distance/physical distance.

c Correlation between marker order and their physical positions.

d Non-pseudoautosomal region.

e Pseudoautosomal region.

We were unable to assign 2,946 of the 9,713 SNPs to the linkage map. Of the 7,822 SNPs that passed quality control, 7,510 SNPs were allocated to a confirmed chromosome. Six hundred and fifty two SNPs that had an assigned chromosome were not mapped because a unique map position could not be found and their inclusion based on physical position served to increase the length of linkage map above the defined threshold. There were 91 SNPs with unknown physical position, thus preventing their insertion analysis.

### Comparison with Bovine genome Btau 4.0

**Figure 2:**
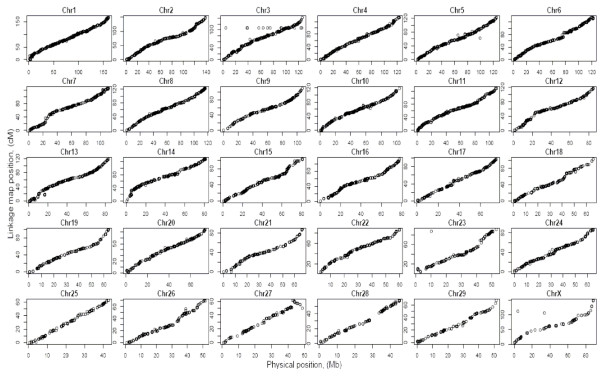
**Comparison of marker linkage map order with positions in the bovine genome sequence assembly (Btau 4.0) **[32].

There was not complete concordance in marker order between the linkage and physical maps (Figure 2). The average Pearson correlation between the order of linkage positions and the physical positions was 0.985 over the genome. Although the correlations were high for the majority of the chromosomes, there were a number of local discrepancies (Additional file 2). Both point discrepancies (e.g. see Figure 2 for chromosome 3) and inversions (Figure 2, chromosome 27, distal region) were observed.

## Discussion

The linkage map presented in this paper is the most dense map to date for cattle; the relatively high number of informative meioses per available SNP represented in the 10K SNP panel is greater than that reported by Snelling et al. [16] thus enabling a high degree of marker placement by the mapping software. The number of SNPs that were available from the 10K SNP panel could have been increased further. For example, this could have been accomplished by lowering the allele frequency criterion used to remove any SNP, from 5% to 2%. This would have allowed informative meioses to dictate the placement or rejection of SNPs by the mapping software into the linkage maps. The detection of displaced SNPs was carefully monitored. Some displaced SNPs had formed clusters with small genetic distances between them but the cluster was placed further than the established threshold of 20 cM from either the most-distal or most-proximal marker of any other linkage group. The success rate for identifying these SNPs relied on the information content of each one of the markers. We set more stringent criteria for marker placement than was previously published [16]; that is, we only accepted clusters with LOD scores above 15 and where at least two microsatellites belonged to the linkage group. Further, we did not allow linkage to any other groups. The subsequent placement of orphan and displaced SNPs in other than the originally assigned linkage maps assured us that the methodology utilised in assigning such markers to a chromosome was appropriate. Our autosomal linkage map of 3097.4 cM is very similar in length with the map presented by Ihara et al. of 3,013.5 cM [22]; is longer in 16 and shorter in 13 of the bovine autosomal chromosomes, with an average absolute difference of 8.2 cM per chromosome. The biggest difference in length occurred for chromosome 14, where our map was longer by 23.5 cM. The extra length for chromosome 14 is due equally to extra marker coverage and to expansion in the linkage map. For example, our linkage map for chromosome 14 contains additional markers at the proximal and distal regions of the Ihara et al. [22] linkage map, but the distance between common proximal and distal markers is larger. Of the two markers that mapped proximal to, and the 12 markers mapped distal to common markers with the linkage map of Ihara et al. [22], only one was placed during mapping round 5: Insertion phase, which utilises physical map data (see methodologies section). The positions of all other markers in these two regions were based on linkage information. The genetic positions of the two proximal markers and the 11 distal markers that were placed by linkage information are in concordance with their physical position, except for a cluster of three SNPs. The order of common microsatellite markers that were assigned to our genetic map as well as several other bovine linkage maps [16,18-22] are in complete agreement. Likewise, SNPs common to the genetic map presented by Snelling [16] and our linkage maps are in concordance.

The addition of 6,767 SNPs from the 10K SNP panel to the low-density microsatellite-based maps (LDM) resulted in both expansion and additional coverage of the linkage maps. The expansion was explained by an increase in genetic distance from proximal to distal markers of LDMs. This additional coverage was measured by an increase in genetic distances from the placement of the last SNPs of the 10K SNP panel to the proximal and the distal marker of a LDM. The magnitude of the expansion was of 80.4 cM and the coverage increased by 338.2 cM. The high reliability of the map presented here was made possible because of a high accuracy of genotyping, thorough pre-screening of the genotypic data for inconsistencies (mis-inheritance, departure from HWE, low allele frequency and less than 10 informative meioses), relatively high numbers of informative meioses and the ability to place orphan and displaced SNPs. Hence this map will be useful to monitor the bovine genome assembly. Using the approach applied by Breen et al. [28], a map resolution of 0.80 cM between autosomal markers could be obtained from an average of 366.9 informative meioses. For our 3,097.4 cM autosomal linkage map the number of markers that could potentially be placed to unique positions is 3,872. Our autosomal linkage map has 3,078 unique marker positions and should be considered as not fully saturated. The average Kosambi distance is lower than that presented by Snelling et al. [16]. However, the coefficient of variation (CV) is greater, indicating that our linkage maps have a higher proportion of marker clusters (Table 2), (Figure 1). The insertion of an otherwise un-mapped SNP by using its physical position is the most probable cause for the increased value in observed CV. An un-mapped SNP that belongs to a scaffold that already includes a mapped SNP(s) is not expected to increase genetic distances because it creates a cluster rather than a singleton.

The observation that chromosome size increases the average recombination rate was consistent with other studies [30,31]. The average recombination distance of 1.25 cM per Mb was similar to the value of 1.19 reported by Kong et al. in humans [31] and approximately twice that of the value of 0.63 found in mice (Shifman et al. [30]). Based on the bovine assembly Btau 4.0 [32], the total physical length from first proximal to last distal markers of our linkage maps was 2.605 Gbp (Table 3). Snelling et al. [17] reported a genome size of 3.1 and 2.9 Gbp estimated from the BAC and sequencing bovine genome project, respectively. Using a physical map of 3 Gbp, the average recombination distance would be approximately 1.1 centimorgans per million base pairs. These inconsistencies also introduce uncertainty in calculating chromosome-wise recombination rates. Inconsistencies between the order of markers in the linkage maps and their physical order (Additional file 2) prevented us from further investigating the recombination distance per physical distance within the chromosome.

The 7K-linkage map presented here has substantially improved on the previously incomplete assignment of SNPs from the 10K SNP panel, and has reordered SNPs that had been wrongly assigned. Thus, our linkage map has shown utility for identifying errors in the current sequence assembly of the bovine genome. In addition, the markers and linkage map will be valuable for fine mapping of QTL [33,34].

The assignment of SNPs to a chromosome from the 10K SNP panel was incomplete and some of their SNPs were wrongly assigned. The assigning and re-assigning of orphan and displaced SNPs to a chromosome and the further placement of these SNPs to unique positions in the linkage during mapping rounds 2–4 was based totally on linkage information. The inclusion of SNPs with up to 50 mis-inheritances in the construction of linkage maps did not have an effect on recombination distances. The markers and linkage map presented in this paper will be useful in the fine mapping of QTL using LD methods [33,34]. However, a number of marker clusters and gaps remain (Figure 1). Further marker development that is being undertaken in the bovine genomics community will ensure that there is greater uniformity and marker density over the genome, which will be beneficial for applications of genomic selection [35]. In addition, the placement by linkage of SNPs from the 10K SNP panel (mapping rounds 2–4) will be useful in identifying inaccuracies in sequence assembly in the bovine genome assembly and in correcting chromosomal assignment for some SNPs from the 10K SNP panel. Approximately 20% of SNPs from the 10K SNP panel were not acceptable for map construction. The major factor for non-acceptance was an allele with a frequency lower than 5%. This probably reflects the origin of the SNPs coming primarily from the sequence of a Hereford cattle and being validated in different populations to the New Zealand Holstein-Friesian and Jersey cattle breeds. That is, this limitation could be a reflection of the breed origin of the SNP. The use of breed-specific SNPs and the knowledge of the physical position of SNPs are two aspects that should not be overlooked. Structural discrepancies observed between the order of the markers in the linkage map, and their physical position (Additional file 2), could be attributed to spurious information in the bovine assembly Btau 4.0 [32]. In the opinion of the authors, at the present time, the number of informative meioses has more weight in the acceptance of the linkage position of the markers than their physical position.

## Conclusion

Using a unique animal resource, 7066 bovine genetic markers were positioned in our linkage map. Approximately 90% (6767 out of 7510) of the SNPs that passed quality control testing from the 10K SNP panel were placed on the linkage map (Additional file 3). The marker positions in the linkage maps are in good agreement with the physical positions obtained using Btau 4.0 of the bovine genome. The information from this linkage map has been used to describe patterns of LD in the bovine genome [36]. Additionally, it will support further genetic analysis of important economic traits in cattle and will help to resolve challenges encountered in the assembly of the bovine genome. The linkage map is not fully saturated, and thus the addition of more markers would be valuable.

## Methods

### Population

An outbred F_2 _experiment of Holstein-Friesian and Jersey cattle breeds was undertaken in New Zealand to identify QTL and genes affecting dairy production [37]. The experiment consisted of 817 F_2 _females, 796 F_1_dams, 6 F_1 _sires and 60 F_0 _males (Additional file 4). All sires of F_1 _dams and F_1 _sires are represented in the set of 60 F_0 _sires. There were no matings between individuals that shared a sire.

### Genotyping

In total, 1679 animals (male F_0_, as well as all F_1 _and F_2 _animals) from the experiment were genotyped by external laboratories according to standard practices for fluorescent dye-labelled primers, utilising Applied Biosystems 3100 genetic analysers (Australian Genome Research Facility, Melbourne, Australia and GeneMark™, Hamilton, New Zealand) for 294 microsatellites; three milk protein haplotypes: 1) Alpha s1 casein (CSN1S1) formed by A_CAS_41_26 and AS_CAS_192; located at 6517 and 17807 base pairs (bp) of locus X59856 (accession number X59856, AJ812028) respectively, 2) Kappa casein (CSN3) formed by K_CAS_148, located at 5345 bp of locus X14908 (accession number X14908) and 3) Beta casein (CSN2) formed by B_CAS_37, B_CAS_67, B_CAS_106 and B_CAS_122, located at 690, 8101, 8219, and 8267 bp of locus X14711 (accession number X14711) respectively [38], two gene-based SNPs (The non-conservative K232A substitution in the DGAT1 gene [39,40] and the F279Y SNP, which is a substitution in the transmembrane domain of the GHR gene [41]) and the 10K SNP panel. T six F_1 _sires were screened for approximate 500 microsatellites. Where four out of six sires were heterozygous, the markers were used. The 10K SNP panel was genotyped 12 months later than the other markers.

### SNP Quality Control

Before undertaking construction of high-density bovine linkage maps, SNPs from the 10K SNP panel were screened for segregation distortion by HWE [42] and mis-inheritance. A SNP showing any of the following criteria: departure from HWE (P-value less than 0.001), more than 50 records of mis-inheritance (inheritance had previously been confirmed from the microsatellites), an allele with a frequency lower than 5% in the F_0 _and F_1 _populations, or less than 10 informative meioses, was deleted from further analysis. The remaining SNPs that passed quality control testing for map construction each had at least one case of mis-inheritance.

### Pedigree Structure

The linkage mapping utilized 1679 individuals from the F_2 _design described by Spelman et al. [37]. All informative meioses for the autosomal maps are male and thus the maps are male-specific. The same is true of the pseudoautosomal part of the sex chromosome.

The non-pseudoautosomal part of the sex chromosome was constructed differently; it utilized maternally-derived genotypes (F_1 _dam) and was therefore a female-specific map. The F_2 _daughters' genotypes were comprised of maternally-derived alleles as well as paternally- (F_1 _sire) inherited haplotypes. The maternally-inherited alleles were derived by subtracting the maternally-inherited haplotypes from the progeny genotypes as follows. Because recombination is not possible for the haploid sex chromosome in males, these maternally-inherited haplotypes represented entire (non-pseudoautosomal) chromosomes. This in turn enabled the maternally-inherited haplotype to be determined in the F_2_. As for their F_2 _daughters, the F_1 _dams' chromosome-long haplotypes were known. This is because their sires (the F_0 _maternal grandsires) were genotyped. Therefore the F_1 _dams' phases were known, increasing the ability to observe recombination events amongst their F_2 _offspring. Our linkage map is based on a two-generation pedigree and it could be further enhanced using a three-generation pedigree. The number of animals involved in the pedigree structure, number of markers involved in map construction and limitations in hardware capability limited the use of a three-generation pedigree.

### Construction Low-density microsatellite based linkage map (LDM)

There were five rounds of mapping. The first one used limited marker data (294 microsatellites, three milk protein haplotypes and two gene-based SNPs) and hence resulted in a low-density microsatellite-based linkage map. Subsequent rounds incorporated SNPs from the 10K SNP panel and enabled the construction of high-density linkage maps.

### Mapping round 1

**Figure 3:**
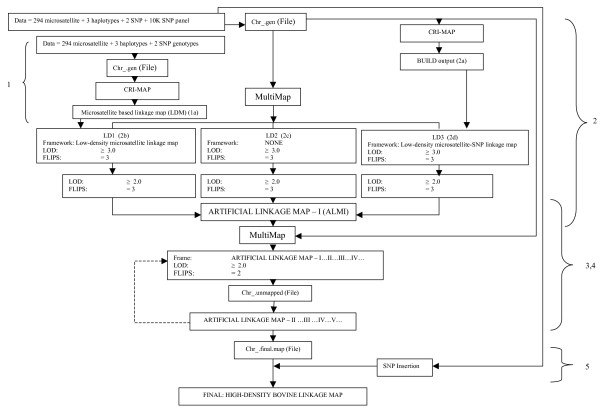
**Mapping flow chart**. (1) Construction of low-density linkage map. (2) Construction of ARTIFICIAL LINKAGE MAP (ALMI). (3) and (4) Additional SNPs mapping loops. (5) SNP insertion phase. LDM: Low-density microsatellite based linkake map. LD1: Low-density microsatellite linkage map. LD2: Low-density SNP linkage map. LD3: Low-density microsatellite-SNP linkage map. LOD: Log of Odds.

The LDM was constructed based on 294 microsatellites, three milk protein haplotypes and two gene-based SNPs (Figure 3(1a)). Construction of the map was done using the software package CRI-MAP V. 2.4 – Build option [43,44]. Modifications were done locally to the software to allow it to run on a 64-bit Opteron with 32 GB physical memory with a swap partition of 10 GB. No user memory limit was enforced. The CRI-MAP Chrompic Option [43,44] was used to remove unlikely double recombinants over a distance of 5 cM. The linkage map created in this initial round was used as framework map in mapping round 2 (Figure 3(2b)).

### Construction of High-Density Bovine Linkage Maps

The 10K SNP panel did not have complete assignment of SNPs to a specific chromosome. Of the 7822 SNPs available from the 10K SNP panel, 1189 (orphan SNPs) were not initially assigned to a chromosome. Using the mapping information from mapping round 1, CRI-MAP V.2.4 (TWOPOINT option) [43,44], 1053 of these orphan SNPs were assigned to a chromosome. The criteria were: a likelihood of odds (LOD) threshold greater than 15 with at least two microsatellites belonging to the same linkage group and no other significant linkage to an alternative chromosome. In addition to CRI-MAP V.2.4 [43,44], the expert system software package MultiMap [45] was used to create the high-density bovine linkage map.

The MultiMap [45] parameter flip was evaluated by using different values. The optimum values for the flip parameter for these types of dense linkage maps are above three. When parameter flip values over three were used for the bovine chromosome 29 with 144 markers, it was found to be time-consuming, (from four-fold to 196-fold for flips 4 to flips 6, respectively) or halted when the parameter flip was set to seven. Our ability to support the final placement of markers in linkage maps with the use of a value higher than three for the parameter flips was prevented by the constraints of our computer hardware.

### Mapping round 2

For each bovine chromosome, three low-density linkage maps were constructed: 1) low-density microsatellite linkage map (LD1) (Figure 3(2b)), 2) low-density SNP linkage map (LD2) (Figure 3(2c)), and 3) low-density microsatellite-SNP linkage map (LD3) (Figure 3(2d)). This mapping round was undertaken to map 7686 SNPs that had been physically assigned to a chromosome. MultiMap [45] constructs comprehensive maps by using framework maps that can either be built by the program or supplied by the user. For LD1, the LDM from mapping round 1 was used as the framework. No framework map was used for LD2. For LD3, the map constructed by CRI-MAP V. 2.4 – Build option [43,44] (2a) was used as the framework. To enter a linkage map, the position for the SNPs had to exceed a LOD score of three with the Flips Option set to three. After all qualifying SNPs were mapped; the LOD score for SNP acceptance was lowered to two, thus allowing additional markers to be positioned. LD1 maps will always have all makers from LDM, plus additional SNPs from the 10K SNP panel.

The low-density linkage maps (LD1–LD3) comprise a mix of common markers (microsatellites as well as SNPs) and differ from each other only in SNPs from the 10K SNP panel. The three separate low-density maps (LD1, LD2 and LD3) were integrated into one linkage map termed ARTIFICIAL LINKAGE MAP – I (ALMI). The integration procedure was performed observing the following rules: markers that appeared in more than one of the three linkage maps were anchored; markers that occurred only in one of the low-density linkage maps were integrated into the ALMI, retaining their original order with respect to other markers within their own low-density linkage map. The resulting ALMI had a greater number of markers than the individual low-density linkage maps (LD1–LD3). There were no inconsistencies in SNP order among the three different low-density maps. In some cases, the integration of a marker was difficult due to the ambiguous positions where it could be placed. However, this had no impact on the linkage map because MultiMap [45] was able to resolve the order in the subsequent runs during mapping round 3.

### Mapping round 3

SNPs not mapped during mapping round 2 were brought into the linkage map using an iterative procedure with the AMLI used as the framework map. To enter the map, the position for the SNPs had to exceed a LOD score of two with the Flips Option set to two. The proposed placements suggested by MultiMap [45] for the remaining unmapped SNPs were tested and the SNP was placed if the Kosambi distance was equal or less than 0.5 centimorgans (cM) to the nearest marker. In some instances, a subsection of 20 SNPs in the region of a possible location was created as a framework map; MultiMap [45] was then able to place such SNPs. This methodology was continued until: a)- no further SNPs were placed into a unique position, b)- Proposed alternative placements suggested by MultiMap [45] numbered greater than three, or c)- a SNP was placed at both ends of a chromosome. During this mapping phase, several SNPs initially assigned to a specific chromosome were placed more than 20 centimorgans (cM) from either the most-distal or most-proximal marker. These SNPs (955 displaced SNPs) were removed from the linkage group as the linkage information indicated that they had been physically assigned to the wrong chromosome. A total of 779 of these SNPs were successfully assigned to a new chromosome using the previously described method in assigning an orphan SNP to a chromosome.

### Mapping round 4

This round consisted of mapping the 779 re-assigned SNPs, followed by one further round of mapping for all SNPs from the 10K SNP panel that had not been placed during mapping round 3. The mapping criteria were same as in mapping round 3.

### Mapping round 5: Insertion phase

The remaining unmapped SNPs from the 10K SNP panel after mapping round 4 were inserted into the linkage map at a position where they were neighbouring the SNP with the closest physical position. Initially, the physical positions for SNPs were obtained from the bovine assembly Btau_3.1 [46]. The final physical positions used in the insertion phase were from the bovine assembly Btau_4.0 [32]. The insertion of SNPs was done from proximal to distal orientation. No attempt was made to study consequences of a SNP insertion in the opposite direction. A SNP was retained in the linkage map if its insertion increased the length of the linkage map by less than 0.5 cM, or the Kosambi distance with the nearest markers was equal or less than 0.5 cM.

### Recombination distance per physical distance

Recombination distances and marker physical positions (obtained from bovine genome assembly (Btau 4.0) [32]) were used to estimate recombination distances per physical distances. Pearson correlations were calculated between marker order and their physical positions.

## Authors' contributions

JA construction of bovine autosomal chromosomes 2–13, 15–29 linkage maps and writing of manuscript. MK provided bioinformatics support. PF construction of bovine sex chromosome linkage map. WC construction of bovine chromosomes 1 and 14 linkage maps. RS initial data cleaning and analysis of results.

## Supplementary Material

Additional file 1 **Unmapped SNPs from the 10K SNP panel**. Excel spreadsheet containing unmapped SNPs from the 10K SNP panel and the reason why the marker was not mapped. Click here for file

Additional file 2 **Linkage map**. Excel spreadsheet containing linkage maps of bovine chromosomes. Data include chromosome, marker name, and marker reference: DataBase and ID, marker type, informative meioses, Kosambi distance, position in chromosome in centimorgans, marker physical position (Bovine genome sequence assembly (Btau 4.0) [32], information on marker mapping round (1–4) or its insertion into linkage maps by means of physical position and number of genotypes removed per marker due to unlikely double recombinants. Click here for file

Additional file 3 **Summary final SNP status from the 10K SNP panel**. Excel spreadsheet containing total numbers of mapped, available, removed for quality control reasons, and unmapped SNPs from the 10K SNP panel. Click here for file

Additional file 4 **Information on pedigree structure**. Microsoft Word document presenting number of animals forming the pedigree structure utilized as the animal population in the map construction. Click here for file
