# Supplementary material for: A high density linkage map of the bovine genome
Source: BMC Genet. 2009 Apr 24;10:18. doi: 10.1186/1471-2156-10-18 (PMC2680908; doi:10.1186/1471-2156-10-18)
Supplement: Additional file 4 — Information on pedigree structure. Microsoft Word document presenting number of animals forming the pedigree structure utilized as the animal population in the map construction. [file 1471-2156-10-18-S4.doc]

F2 F1 F0

6 Sires^a^

6 Sires

817

60 Sires^b^

796 Dams

a These six paternal grandsires were part of group of maternal grandsires

b Maternal grandsires
